# Bioreceptors’ immobilization by hydrogen bonding interactions and differential pulse voltammetry for completely label-free electrochemical biosensors

**DOI:** 10.1007/s00604-024-06738-x

**Published:** 2024-10-14

**Authors:** Adaris López-Marzo, Marta Mas-Torrent

**Affiliations:** Institut de Ciència de Materials de Barcelona, ICMAB-CSIC, Campus UAB, 08193 Bellaterra, Spain

**Keywords:** Hydrogen bonding, Differential pulse voltammetry, Label-free immunosensors, Hepatitis B virus, Gold electrodes

## Abstract

Label-free electrochemical biosensors show great potential for the development of point-of-care devices (POCDs) for environmental and clinical applications. These sensors operate with shorter analysis times and are more economic than the labelled ones. Here, four completely label-free biosensors without electron transfer mediators were developed for hepatitis B virus (HBV) detection. The approach consisted in (i) the modification of gold surfaces with cysteamine (CT) or cysteine (CS) linkers, (ii) the subsequent antibody (Ab) immobilization, either directly by hydrogen bonding (HB) interactions or by covalent bonds (CB) using additional reagents, and (iii) measuring the biosensor response by electrochemical impedance spectroscopy (EIS) and differential pulse voltammetry (DPV). The electrode surfaces at each stage of the modification process were characterised by X-ray photo-electron spectroscopy (XPS) and atomic force microscopy (AFM). The combination of Ab immobilization by HB with the DPV analysis displayed improved repeatability, lower interference to serum matrix and similar limits of detection and quantification than the traditional biosensors that immobilize the Ab via CB and use EIS as readout technique. The Ab immobilization by HB is shown as a simple, efficient and low-cost alternative to CB ones, while DPV was faster and showed better performance than EIS. The CT-HB biosensor displayed the lowest limits of detection and quantification of 0.14 and 0.46 ng/mL, respectively, a 0.46–12.5 ng/mL linear analytical range, and 100% of recovery for 1/10 human serum media during HBV surface antigen detection by DPV. Even, it preserved the initial sensing capability after 7 days of its fabrication.

**Graphical abstract:**

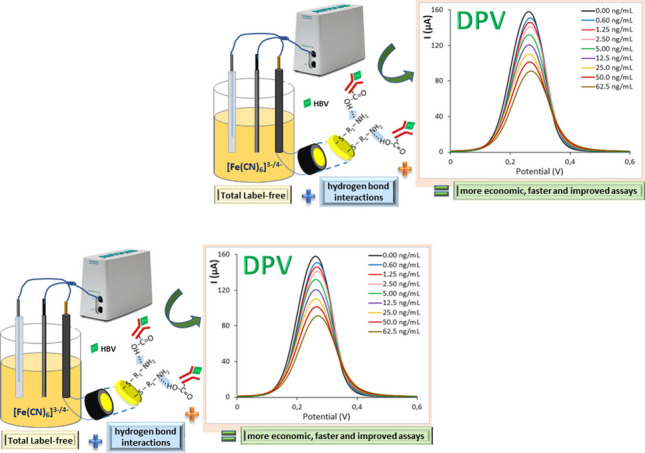

**Supplementary information:**

The online version contains supplementary material available at 10.1007/s00604-024-06738-x.

## Introduction

Electrochemical biosensors are helpful tools for detection and quantification of analytes of clinical and environmental interest [1, 2]. They constitute a low-cost, fast and reliable alternative to the traditional time-consuming and expensive analytical methods such as high-performance liquid chromatography (HPLC) or enzyme-linked immunosorbent assay (ELISA). Electrochemical biosensors have shown potential for the development of point-of-care devices (POCDs) due to their easy portability, economic cost and immediate results (sample-in and answer-out), allowing the decentralization of the analyses with devices that can be operated by a non-specialised person [1, 3, 4]. Specially, label-free electrochemical biosensors are suitable to implement in POCDs because the second bioreceptor with the electrochemical tag is eliminated, which reduces the time and cost of analysis compared to the labelled assays [5, 6]. Therefore, in label-free electrochemical biosensors, the biological recognition of the target analyte by the first bioreceptor anchored to the electrode surface is directly transduced in changes of the electrode electrical properties. In the manufacturing of label-free electrochemical biosensors, gold is the principal material used as working electrode due to its multiple advantages [7]. The gold high conductivity enables the charge transfer from the solution to the electrode surface, which is essential for achieving a fast response and enhanced biosensor performance. Gold is inert, biocompatible and presents well-established methodologies for surface biomodification. The routes of gold surface biomodification are based on the preparation of molecular self-assembled monolayers (SAMs) [3, 8]. For this purpose, a bifunctional molecule (linker) bearing a thiol end group to bind with the gold surface, and a carboxylic or amino terminal group to form a covalent bond (CB) with the bioreceptor molecule (antibody (Ab), DNA, enzyme, etc.) is commonly used [9, 10]. The formation of a CB with the biocaptor molecule is mostly addressed by the activation of the COOH groups with ethyl(dimethylaminopropyl)carbodiimide/N-hydroxysuccinimide (EDC/NHS) leading to an amide bond (i.e. peptide bond) [3, 4, 6] or the activation of the NH_2_ groups with glutaraldehyde giving an imine bond. However, it should be considered that intermolecular non covalent hydrogen bond (HB) interactions are known to play an important role in nature. They are responsible for holding the 3D structure of proteins and other macromolecules without biological activity [11] and they are associated with the development of supramolecular chemistry [12]. Many HB interactions are established between amino acids of peptides, whose strength lies in the range 40–50 kJ/mol [11, 13].

In label-free electrochemical biosensors an external redox probe such as [Fe(CN)_6_]^3−/4−^ is used as electrochemical tracer to directly transduce the biological recognition of the target analyte into changes of the electrode electrical properties. Likewise, electrochemical impedance spectroscopy (EIS) is the electrochemical technique of preference to accomplish this analytical detection due to its high sensitivity in detecting electrical changes at the electrode surface as consequence of variations in the electrode composition [2, 3, 6, 14–16]. However, EIS presents some drawbacks. First, it requires of several minutes (3–5 min) to record one complete data set since a wide frequency range is scanned. Further, EIS needs to adjust the measurement data to an equivalent circuit to calculate the electron transfer resistance (R_CT_) [16, 17]. In addition, EIS displays a low repeatability compared to other electrochemical techniques [18]. There are reports of other label-free analyses using direct current (DC) electrochemical techniques such as differential pulse voltammetry (DPV) [5, 19–21], amperometry [22] and square wave voltammetry (SWV) [23, 24]. However, these techniques need additional materials, such as metal–organic frameworks [5], conducting polymers [25, 26], carbon-based materials [20–24, 26, 27] and metal NPs [21–27], modifying the electrode surface to enhance the electron transfer (electron transfer mediators), or for signal generation itself (electroactive indicators) to produce the quantifiable signal that proportionally changes with the analyte concentration [19, 28]. There are also many label-free biosensors using DC electrochemical techniques [5, 19–23], and even EIS [14, 29], using electron transfer mediators or electroactive indicators as surface modifiers on gold [5, 23, 29], ITO [19, 20] and carbon [14, 21, 22] electrodes.

Hepatitis B virus (HBV) is the most spread viral hepatitis around the world. In 2019, 296 million people were living with HBV [30]. It is highly resistance and stable outside the body and the most infectious of the hepatitis viruses, producing irreversible damages in the liver (cirrhosis, fibrosis, liver failure, cancer) and even the patient death [22, 28, 31]. Its detection is crucial to apply adequate treatments toward the disease cure and patient survival. Thus, the development of detection strategies of easy implementation in POCDs for HBV diagnosis and treatment is key.

Here, completely label-free electrochemical biosensors to detect the HBV surface antigen (HBsAg) were fabricated without adding electron transfer mediators as electrode modifiers and using the combination of HB interactions for Ab immobilization and DPV as measurement technique. We prepared four biosensors based on two Au-linkers and two methods for Ab anchoring. We used linkers with different terminal groups (COOH in cysteine (CS) and NH_2_ in cysteamine (CT)) to promote different mechanisms of Ab immobilization for CB formation and HB interactions. Thus, the influence of the linker-Ab binding on the sensor’s response was explored. The analytical detection was carried out by DPV and EIS, simultaneously. This new strategy of combination of HB for Ab immobilization and DPV as analytical technique provides biosensors with higher repeatability, better recoveries in serum matrix and similar limits of detection and quantification than those using the traditional Ab immobilization by CB and EIS technique for the analytical determination. This work elucidates the Ab immobilization by HB interactions as an efficient alternative route for biosensor manufacturing that does not require additional chemical reagents and simplifies the functionalization steps for analysis. In addition, the as-fabricated label-free sensors operate using the fast and easy processing DPV technique. The CT-HB biosensor exhibits a low limit of detection of 0.14 ng/mL (5.8 pM) for HBsAg, a relevant biomarker of HBV disease that constitutes an important global health problem. Additionally, the CT-HB biosensor preserved its initial sensing performance after 7 days of its fabrication.

## Material and methods

### Reagents and materials

Potassium ferricyanide (III) (K_3_[Fe(CN)_6_]), potassium hexacyanoferrate (II) trihydrate (K_4_[Fe(CN)_6_]⋅3H_2_O), cysteine (CS) 98%, cysteamine (CT) 95%, glutaraldehyde (GA) solution (50 wt. % in H_2_O), *N*-(3-dimethylaminopropyl)-*N*′-ethylcarbodiimide (EDC), *N*-hydroxysuccinimide (NHS), bovine serum albumin (BSA), human serum from human male AB plasma and phosphate buffer saline (PBS, 0.01 mol/L, pH 7.4) were acquired from Sigma-Aldrich-Merck (Germany). Sulphuric acid (H_2_SO_4_) 98%, ethanol absolute 99.9%, potassium chloride (KCl) and sodium hydroxide (NaOH) were purchased at Chem-Lab (Belgium).

The mouse monoclonal antibody anti-hepatitis B Virus surface antigen (HBsAb, ab8333) and the recombinant hepatitis B virus surface antigen (HBsAg, ab193473) were purchased from Abcam (UK). Polycrystalline gold working electrodes (disk diameter 2 mm and area 3.14 mm^2^), Ag/AgCl reference and platinum wire counter electrodes were purchased from PalmSens (Netherlands), as well as alumina particles powders (Al_2_O_3_, 0.3 and 0.05 μm) and the polishing pads.

All the solutions were prepared in ultrapure Milli-Q water (18.2 MΩ⋅cm at 25 °C and total organic carbon < 5 ppb).

### Equipment and measurements

Cyclic voltammetry (CV), differential pulse voltammetry (DPV) and electrochemical impedance spectroscopy (EIS) measurements were accomplished in a cell with a three-electrode system using the NOVA 2.1.4 program and the PGSTAT128N potentiostat from Metrohm (The Netherlands). All the electrochemical measurements with the modified electrodes (both the final biosensor electrodes and the biofunctionalized electrodes at any intermediate stage of the biomodification process) were carried out in 0.01 M PBS pH 7.4 with 25 mM [Fe(CN)_6_]^3−/4−^ used as electrochemical tracer. CV was conducted from − 0.4 to 0.8 V at 50 mV/s scan rate. DPV was performed in the range of 0 to 0.6 V with 0.01 V step and 0.025 V amplitude pulse. EIS was carried out applying a potential of 0.15 V and 0.01 V amplitude over a frequency range of 1–100 000 Hz. The R_CT_ values of the Nyquist plots were estimated using the equivalent Randles circuit [16].

For X-ray photo-electron spectroscopy (XPS) and atomic force microscopy (AFM) analyses, the samples were prepared on Au-coated glass substrates of 1 × 1 cm. The Au-coated glass slides were prepared by template stripping, modifying a previous methodology, to generate very flat surfaces suitable for AFM analysis [32]. The XPS spectra were recorded at room temperature on a SPECS PHOIBOS 150 hemispherical analyser (SPECS GmbH, Berlin, Germany) using a monochromatic excitation source Al K alpha radiation (1486.74 eV) and operating at 300 W. All the XPS spectra were calibrated with respect to Au peak. In the deconvolution analysis of the high-resolution core level spectra, a Gaussian–Lorentzian line-shape was used to adjust the components, and Shirley subtraction was applied to the background. The AFM topological images were acquired in tapping mode with a Nano-Observer AFM (from Concept Scientific Instruments) using silicon tips of 1 N/m spring constant and 60 kHz of resonance frequency. A surface of 2 × 2 μm^2^ was scanned for each sample at four different regions. Gwyddion 2.62 software was used for the image processing and the estimation of the square mean roughness (RMS) and the height in the AFM surface images.

### Procedures

#### Cleaning and activation of gold electrodes

The optimized cleaning procedure of gold electrodes embraced a combination of mechanical, chemical and electrochemical treatments. First, the gold electrodes were carefully polished by hand making 8-shape circular rotations on mastertex microfiber pads with alumina suspensions (2.4 mg/5 mL) of 0.3 and 0.05 μm during 3 min each time. Then, the electrodes were sonicated in 5 mL of ethanol followed by 8 mL of Milli-Q water during 5 min each. Afterwards, the gold electrodes were electrochemically treated by CV (20 sweeps from − 0.1 V to 1.5 V at 50 mV/s scan rate) in 0.5 M H_2_SO_4_ solution previously degassed with N_2_.

#### Biofunctionalization of the gold electrode surface and biosensors preparation

**Fig. 1:**
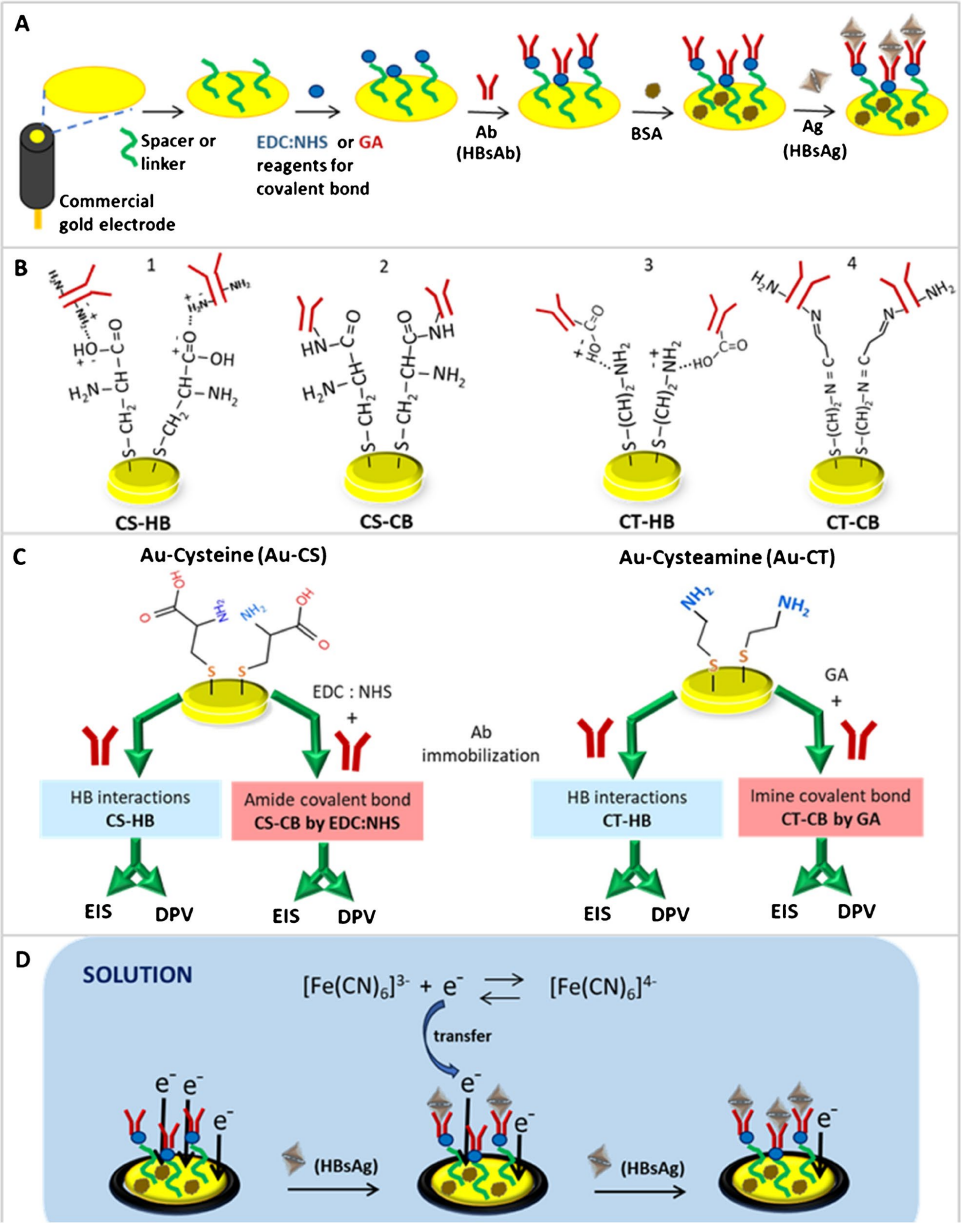
Scheme of the four label-free electrochemical immunosensors developed on gold electrode platforms for HBV detection. **A** General procedure used for gold electrode biofunctionalization via covalent bond formation. **B** Chemical structure of the final Au-Linker-Ab platforms obtained for each immunosensor. **C** Representation of the four immunosensors designed combining CS or CT linkers with HB or CB Ab immobilization procedures, studied with EIS and DPV electrochemical methods. **D** Working principle of the label-free biosensors

After cleaning and activation, the gold electrodes were biofunctionalized as illustrated in Fig. 1A. CT or CS were used as spacer for producing S–Au bonds with the gold electrode surface. For that purpose, 20 μL (1 mM) of CT or CS prepared in NaOH alkalinized (pH 9.5) and N_2_ degassed water were deposited on the top of gold surface and incubated overnight (18 h) at 4–7 °C in a humid chamber. Following, 10 μL (25 μg/mL) of the HBsAb was directly deposited on the surface during 1 h under agitation (60 rpm) for their immobilization by HB interactions. Then, the electrodes were blocked with 20 μL (0.05%, w/v) of BSA by incubating them during 20 min at 60 rpm. Washing steps with PBS were carried out after each modification process. This process led to the CT-HB or CS-HB biosensors (Fig. 1B, 1 and 3).

**Fig. 2:**
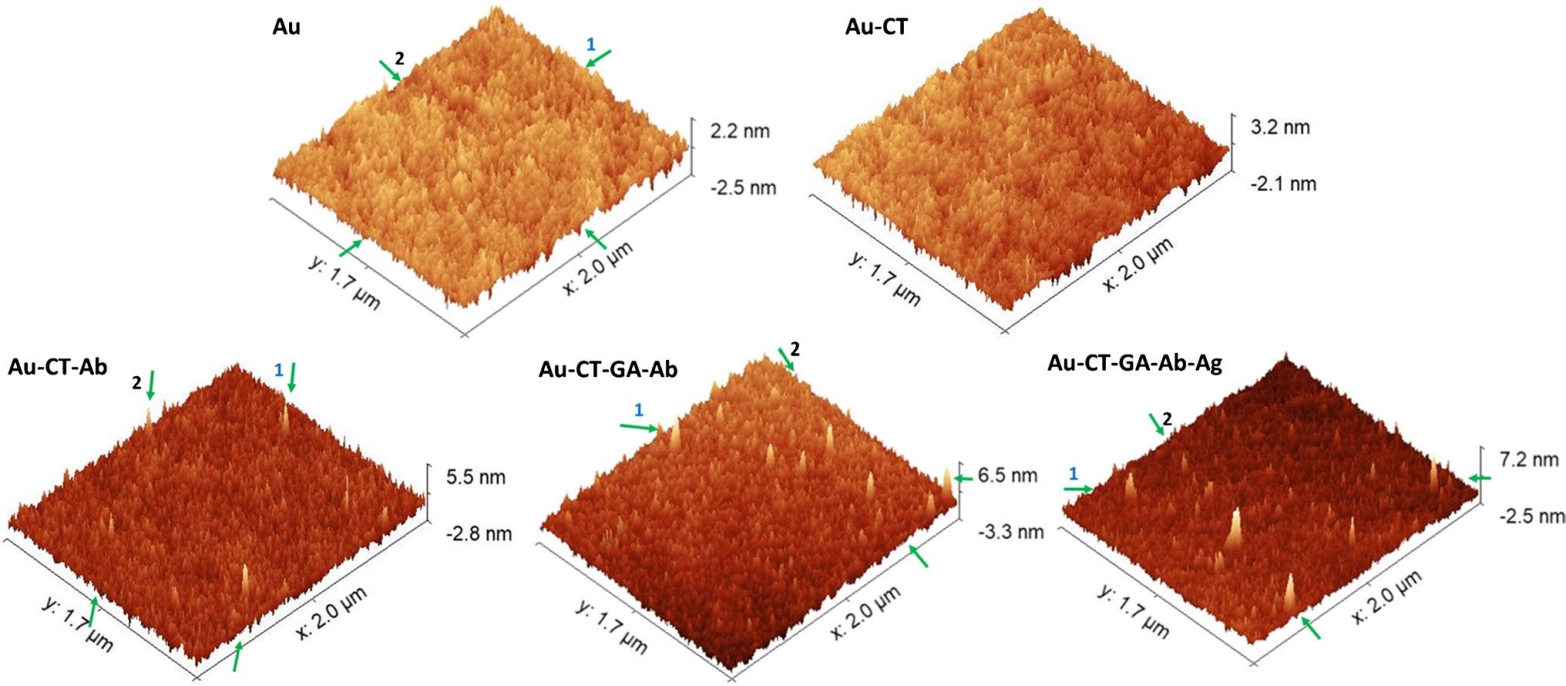
AFM topological images of unmodified Au and Au-CT-modified surfaces. The opposite green arrows label the imaginary line between two points used to obtain the height profiles. An Ag concentration of 0.6 ng/mL was used in the Au-CT-GA-Ab-Ag surface

The Ab immobilization by CB required an additional step for the imide or amide bonds formation (Fig. 1B, 2 and 4).

**Fig. 3:**
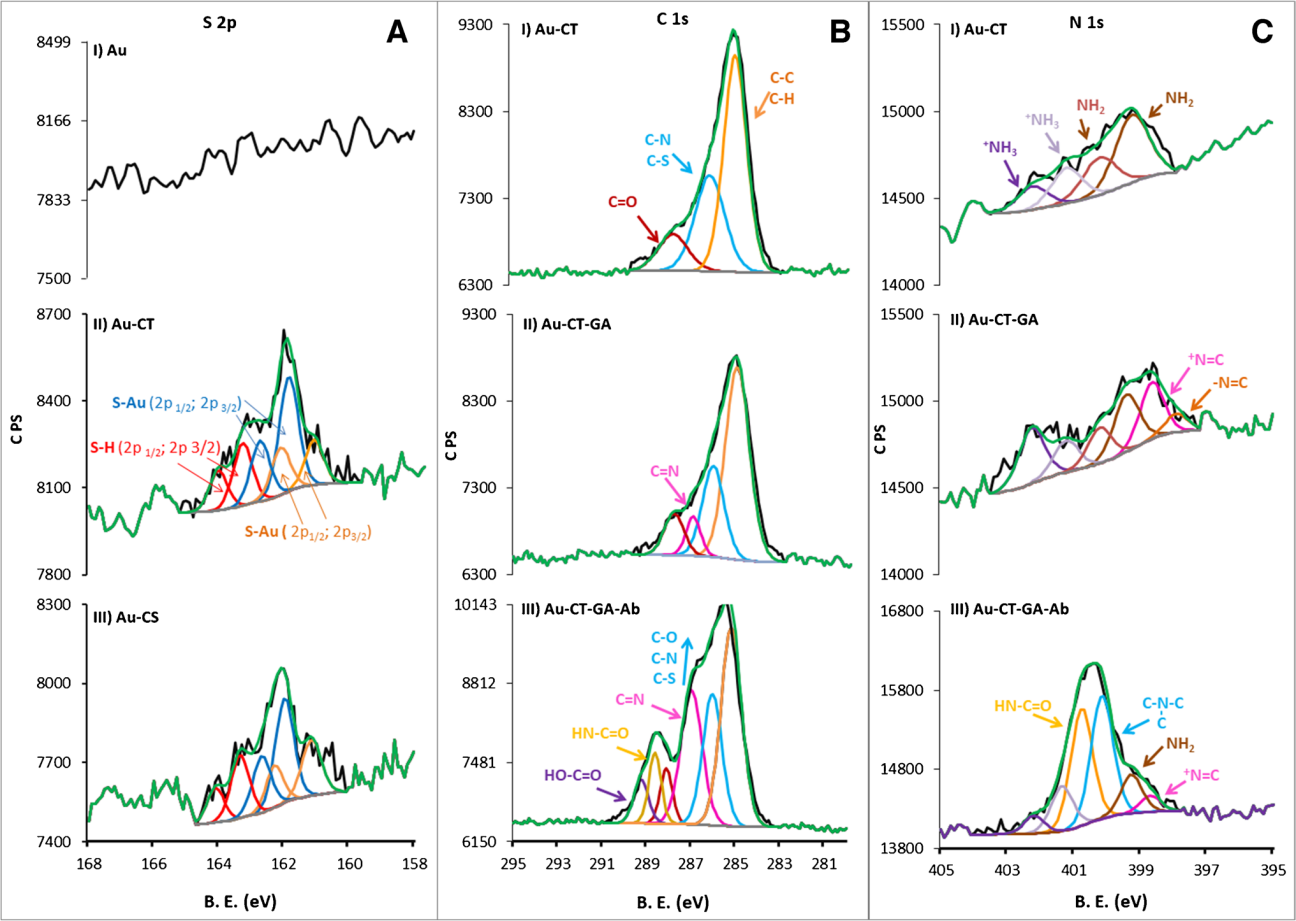
**A** High-resolution XPS spectra of the S 2p core levels for Au, Au-CT and Au-CS platforms, and the C 1 s (**B**) and N 1 s (**C**) core levels for the Au-CT, Au-CT-GA and Au-CT-GA-Ab systems

**Fig. 4:**
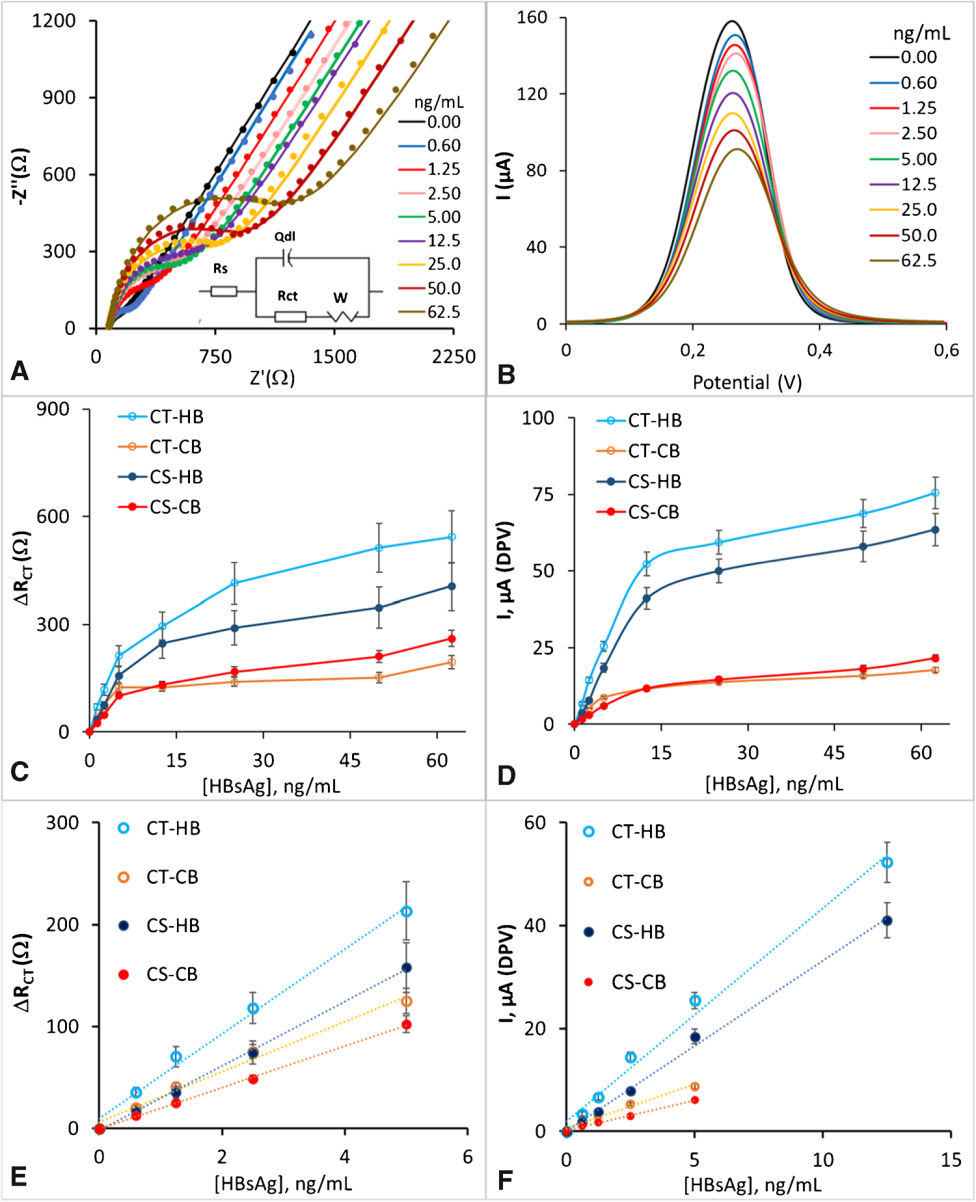
Typical variations in the EIS (**A**) and DPV (**B**) profiles when increasing concentrations of HBsAg are added to the CT-HB-based biosensor. The insert in **A** represents the equivalent Randles circuit. Calibration curves were obtained measuring EIS and DPV during the analysis of increasing HBsAg concentrations for the four-biosensor platforms (**C** and **D**). Linearity study (**E** and **F**) at low Ag concentrations. Each concentration point corresponds to at least four independent replicates

- CS-CB: Immobilization of the Ab by an amide CB between the carboxylic terminal group of the CS and the NH_2_ group in the lysine amino acid of the Ab structure (Fig. 1B and 2). After the anchoring of the CS molecule to the gold surface, the electrode was immersed in 1 mL of a 50 mM solution of EDC: NHS (50:50) and left to react during 20 min.
- CT-CB: Immobilization of the Ab by an imine CB between the terminal NH_2_ groups of the CT and the lysine amino acid in the Ab with the ended aldehydes of the GA (Fig. 1B, 2, 3 and 4). After the CT binding to the gold surface, the electrode was immersed in 1 mL of a 50 mM solution of GA in PBS solution and left to react for 20 min.

Next, in both cases, the Ab solution was incubated on the electrode surface and blocked with BSA as described above for the HB biosensors.

For the sensing experiments by EIS and DPV techniques, the four biosensors (Fig. 1C) were incubated with 20 μL of HBsAg solutions from 0 to 62.5 ng/mL during 20 min at 60 rpm.

#### Biosensor working principle

Detection mechanism of this biosensor is based on the biological recognition reaction of antigen–antibody and the hindrance that it produces to the transference of electrons from the [Fe(CN)_6_]^3−/4−^ redox probe in solution to the electrode surface [14, 23]. It is a label-free biosensor that detects the transferring of electrons toward the electrode surface product of the redox reaction of the [Fe(CN)_6_]^3−/4−^ happening in solution. If the specific mouse monoclonal antibody anti-hepatitis B virus surface antigen (HBsAb, ab8333) is immobilized on the electrode surface, and the concentration of the recombinant hepatitis B virus surface antigen (HBsAg, ab193473) in contact with the electrode surface increases; then, the amount of antigen binding to the Ab increases too. As consequence of this Ag-Ab reaction, the electrons’ transference between the redox probe [Fe(CN)_6_]^3−/4−^ in solution and the electrode surface decreases, and less current is detected during the measurements by the electrochemical techniques (CV, DPV and EIS) (Fig. 1D).

#### Calibration curves

Aliquots of 20 μL with increasing concentrations of the Ag solutions in 0.01 M PBS (0, 0.6, 1.25, 2.5, 5, 12.5, 25, 50 and 62.5 ng/mL) were incubated for 20 min on gold electrodes prepared as described above. The electrodes were washed with PBS after the incubation with the Ag solution. Next, the electrodes were immersed in 25 mM [Fe(CN)_6_]^3−/4−^/PBS pH 7.4 solution and the electrochemical measures of EIS and DPV were carried out. For the same biosensor platform, at least four calibration curves were obtained as independent replicates of electrodes and days.

#### Matrix effect assays

Human serum sample was selected to evaluate the matrix effect on the response of the CT-HB and CT-CB biosensors, because of it is a fluid commonly used in the hospital’s laboratories to detect and diagnose HBV. In the matrix effect assay, four serials of increasing Ag concentrations from 1.25 to 12.5 ng/mL were prepared in PBS, and human serum was diluted in PBS 1/4, 1/10 and 1/20. Both Ag solutions, in PBS and in diluted human serum, were analysed the same day with the as-prepared CT-HB and CT-CB biosensors. The percentages of recoveries were calculated comparing with the corresponding signal response obtained in PBS.

#### Stability assays

Gold electrodes with the CT-HB biosensors were prepared and stored at 4 °C immersed in 0.01 M PBS pH 7.4. HBsAg solutions of 5 and 10 ng/mL in human serum diluted 1:10 were freshly prepared and analysed with the biosensor fabricated after 0, 3, 7 and 15 days. The percentage of response of the biosensor was calculated over the days in relation to 100% of day zero.

## Results and discussion

The cleaning and polishing of the gold electrodes are a pivotal step for their subsequent biofunctionalization. It ensures in a large part, to achieve the same electrode surface in all the biofunctionalization experiments, endowing good repeatability, high sensitivity and enhanced biosensor performance [7, 8, 10]. The efficiency of eleven strategies tested, from soft to hard cleaning conditions, for optimizing the cleaning process was evaluated by analyzing the intensities of the gold reduction and oxidation peaks in the CV (results not shown). As a result, an optimized cleaning procedure was found, and it was described previously in the “Procedures” section. Figure S1 displays the CV profiles in 0.5 M H_2_SO_4_ of different electrodes cleaned during different days with the optimized procedure. These CVs obtained correspond with the characteristic CV of the clean gold surface [8, 10]. The variability of the intensity of the gold reduction peak observed by CV, and the variability in the R_CT_ monitored by EIS indicated that the selected cleaning procedure was repeatable. The R_CT_ of the clean gold surface is in the range of 40–70 Ω, which agrees with previous reported values [4].

The biofunctionalized surfaces of all the biosensors were prepared using the electrodes treated with the optimized cleaning procedure. A study of the morphology at different stages of the modified gold surfaces was performed by AFM. Topological images in Fig. 2 exhibit a boost in the surface roughness along the functionalization steps. The unmodified gold surface showed a roughness of 0.404 ± 0.011 nm. The formation of Au-CT, Au-CT-Ab, Au-CT-GA-Ab and Au-CT-GA-Ab-Ag SAMs provoked a growth in the surface roughness of 0.500 ± 0.014, 0.917 ± 0.123, 0.752 ± 0.029 and 0.972 ± 0.046 nm, respectively. This sequential boosting in the roughness, in agreement with similar modified gold surfaces, points out the changes happened at the surface as consequence of the functionalization [33, 34]. The profiles of heights between opposite green arrows represented in Fig. 2 for Au, Au-CT-Ab, Au-CT-GA-Ab and Au-CT-GA-Ab-Ag are showed in Figure S2. The peak heights of about 4–7 nm are associated with the Ab anchoring on the surface, as previously reported [33, 35].

The electrochemical characterization by CV, DPV and EIS of each stage of the biofunctionalization process for the CS-based biosensors is illustrated in Figure S3. In general, a detriment of the electrical properties at the electrode surface is registered with each molecular layer anchored to its surface. CV and DPV profiles show a decrease in the current intensity of the [Fe(CN)_6_]^3−/4−^ oxidation peak, as well as a shift in this peak position to higher oxidation potentials at the initial functionalization step (from Au to Au-CS). Additionally, there is an increase in the voltage difference between the oxidation–reduction peaks (ΔE) with the increment of the organic layer thickness, which suggests that the heterogeneous electron transfer at electrode surface is reduced. Accordingly, EIS measurements reflect an increment in the electron transfer resistance with the molecular layers’ thickness. The same trend in the electrochemical properties at gold electrode surface were found for the Au-CT functionalized electrodes (results not shown).

A qualitative analysis of the chemical changes at each stage of the biosensor’s preparation was performed by XPS, analyzing the evolution of the peak components of the C 1 s, N 1 s and S 2p high-resolution core levels spectra for the Au-CT and Au-CS-based surfaces (Fig. 3 and Figure S4, respectively). The spectra in the S 2p region for unmodified gold substrate, Au-CT and Au-CS are displayed in Fig. 3A, I–III. As expected, no sulphur contribution is observed in the unmodified Au substrate. In contrast, after the formation of CT and CS SAM, the S 2p spectra can be fitted to three doublets. One of them corresponds to 2p_3/2_ and 2p_1/2_ of S–H groups of unbound molecules physisorbed on the surface, and the other two 2p_3/2_ and 2p_1/2_ doublets are ascribed to S–Au bonds with different coordination numbers [10, 36]. The assignment of bonds of the peak components and their corresponding orbital binding energies, areas and full width at half maximum (FWHM) is shown in Table S1 [10, 36, 37]. The S–Au contribution is around 73% of the total S signal, indicating favorable formation of covalent bonds between the CT and CS linkers and the gold electrode. The C 1 s core levels of the Au-CT-based biofunctionalized platforms are displayed in Fig. 3B, and their peaks’ assignation and binding energies are shown in Table S2. The peaks at 285.0 and 286.1 eV are ascribed to C–C/C-H and C-N/C-S bonds corresponding to the CH_2_-CH_2_ and CH_2_-NH_2_/CH_2_-S groups of the CT [38–41]. The peak at 287.8 eV corresponds to C = O and is associated with the adventitious carbon of environmental contamination [42]. After the reaction with GA, the C = N contribution is observed in the region at 287 eV (Fig. 3B, II) [40]. Finally, the Ab anchoring resulted in a notable increase in the intensity of the peaks in the C 1 s region, especially of those associated to the binding energy of HO-C = O (289.3 eV) and HN-C = O (288.6 eV) groups, as consequence of the multiple carboxylic and amide groups present in the Ab chains (Fig. 3B, III).

Additionally, a boosting in the peak related to the energy of the C = N group (287 eV) is observed as result of the Ab anchoring through the formation of imine covalent bond. In the N 1 s core level spectra of the Au-CT surface, four peaks appeared at 399.2, 400.2, 401.2 and 402.3 eV ascribed to NH_2_, ^+^NH_3_ at the monolayer and from a multilayer, respectively (Fig. 3C, I) [36, 37, 41]. After the reaction with GA, the transformation of the cysteamine’s NH_2_ terminal groups into imine ones takes place. Accordingly, new signals emerge at 397.9 and 398.6 eV associated to –N = C and –^+^N = C contributions, respectively (Fig. 3C, II) [38, 41]. As observed, the Ab tethering significantly increased the area of N 1 s region, especially toward higher binding energies where two new peaks aroused at 400.1 and 400.8 eV in agreement with tertiary amines and amides present in the amino acid’s chains of the Ab (Fig. 3C, III) [43, 44]. Table S3 summarises the relevant parametres for N 1 s region. For a better understanding of the XPS analysis, the schemes of the principal reactions that take place on the Au-CT surfaces are illustrated in Figure S5.

The Nyquist diagrams and DPV curves of the CT-HB and CT-CB biosensors obtained during the HBsAg detection from 0.6 to 62.5 ng/mL are exhibited in Fig. 4 A, B and Figure S6 A-B, respectively. According to the biosensor working principle (Fig. 1 D), in general, both techniques registered a reduction in the currents on the electrode surface with the increment of Ag bound.

The Nyquist plots from the impedance measurements were fitted with a Randles circuit of the type Rs(Qdl[R_CT_W]), being Rs the resistance of the [Fe(CN)_6_]^3−/4−^ solution, R_CT_ the charge transfer resistance, Qdl the constant-phase-element and W the Warburg impedance (circuit inserted in Fig. 4A). Table S4 shows the values of the Randles circuit components for an experiment during the HBsAg detection using the CT-HB biosensor. The constant-phase-element was used instead of the double layer capacitance to improve the fitting of the Nyquist diagrams achieving a good convergence with the experimental points and low values of *χ*^2^. As observed in the Nyquist plots, at high frequencies the diameter of the semicircle part ascribed to R_CT_ displayed a boosting with the Ag amounts bound to the gold electrode surface as consequence of Ab-Ag reaction, that hindrances the electron transfer between the redox probe in the solution and the electrode, as represented in Fig. 1D. In the low frequency regions, the linear tails of the impedance in the Nyquist diagram suggested a diffusional process leading the mass transfer (Warburg diffusion) toward the electrode surface. Likewise, the DPV curves exhibited a decrease in the current intensity with the increasing of the Ag molecules anchored on the electrode surface, impeding the electron transfer from the redox probe in the electrolyte to the electrode surface. The same behaviour in the EIS and DPV curves was also observed for CS-based biosensors when they were incubated with increasing concentrations of HBsAg (results not shown here).

Figure 4C–F show the HBsAg concentrations detected in PBS pH 7.4 for the four biosensors studied using the two analytical techniques (EIS and DPV). All the biosensors displayed two behaviours over the entire range of concentrations tested. At low HBsAg concentrations, a more sensitive response was found, while at higher HBsAg concentrations the response was less sensitive reaching the signal saturation. In general, the biosensors with the Ab immobilized by HB interactions showed more sensitive signal responses over the entire range of concentrations analysed than those prepared immobilizing the Ab by covalent bond, especially at high HBsAg concentrations (Fig. 4C, D). This could be caused by the fact that when the Ab is immobilised to the electrode by HB, the Ab is directly linked to the Au-spacer without additional spacers in between. This could propitiate the charge transfer toward the electrode and, consequently, provide higher sensitivity. Moreover, comparing the two systems based on hydrogen bonding interactions for Ab anchoring, the CT-HB biosensor showed higher sensitivity than the CS-HB counterpart. This might be related with the less steric impediment of the CT structure, which allows the surface grafting of more molecules and, subsequently, of more Abs. On the contrary, the presence of the COOH group in the CS chain might provide steric impediment in detrimental with the packing of the linker on the gold surface

Although the biosensors with Abs tethered by HB produced higher electrical response compared to those with Abs binding by CB, the variability of the signal using hydrogen bonding was higher compared to covalent bond as demonstrated by EIS and DPV techniques for biosensors with the same linker molecule. Table 1 illustrates the coefficients of variation at 1.25, 2.50, 5.0 and 12.5 ng/mL of HBsAg for all the biosensors studied by EIS and DPV. The data from Table 1 also shows an improvement in the repeatability in the biosensors measured by DPV compared to EIS, which points out a better performance of DPV over EIS regarding precision. Moreover, the coefficients of variation obtained using DPV and EIS for the biosensors with the Ab immobilized by CB are in the order those reported for similar platforms for the detection of HBsAg [28, 45].

**Table 1:** Repeatability and intermedia precision assay. Summary of the coefficients of variation (%) for all the studied biosensors on different electrodes measured over different days at the Ag concentrations of 1.25, 2.50, 5.0 and 12.5 ng/mL

	CT-HB		CT-CB		CS-HB		CS-CB	
ng/mL	EIS	DPV	EIS	DPV	EIS	DPV	EIS	DPV
1.25	28.3	19.3	20.7	12.8	32.3	21.1	17.9	13.9
2.5	25.8	13.4	18.7	8.9	30.2	14.6	14.8	9.6
5.0	26.6	12.6	19.5	9.8	30.8	17.3	16.1	9.1
12.5	25.8	14.7	18.6	8.5	29.1	16.6	15.7	9.5

The linear regression analysis applied to the calibration points at low concentrations displayed a linear dependency in the range 0–5 ng/mL for all the biosensors measured by EIS. When DPV was used, only those biosensors with the Ab immobilized by CB presented a linear response in the range of 0–5 ng/mL. Remarkably, in the biosensors with the Ab immobilized by HB, the linear range was widened to 0–12.5 ng/mL. As indicated by the slope of the linear fitting of the calibration curves, the sensitivity of the electrical property measured by EIS (R_CT_) is about 10 times higher than the current intensity measured by DPV. However, this notable difference in sensitivity does not influence much on the detection and quantification limits of these techniques, which are very similar (Table S5). For instance, for the CT-HB biosensor, the LoD was about of 0.1 ng/mL, and for the CT-CB sensor, it was around 0.3 ng/mL using both techniques. This is because EIS presents a larger variability in the signal response, and DPV an improved repeatability that compensates the lower sensitive of this technique. The CT-HB biosensor among all the biosensors studied, displayed the lowest LoD and LoQ of 0.14 (5.8) and 0.46 (19.2) ng/mL (pM), respectively. Noticeably, this LoD is comparable or even better than other LoDs reported for HBsAg detection with label-free electrochemical biosensors in which electroactive labels for signal generation or electron transfer mediators for signal enhencement were employed (Table S6). It should be considered that 0.15 ng/mL of HBsAg equal to 350 IU/mL [46] and that values of HBsAg < 1500 IU/mL, < 20 000 and > 25 000 IU/mL are associated with different stages of the HBV infection. Therefore, the 0.5–12.5 ng/mL analytical range of the CT-HB biosensor is appealing to monitor serum levels of HBsAg in the HBV diagnosis and follow the treatment efficiency in chronic hepatitis B [47–49].

**Fig. 5:**
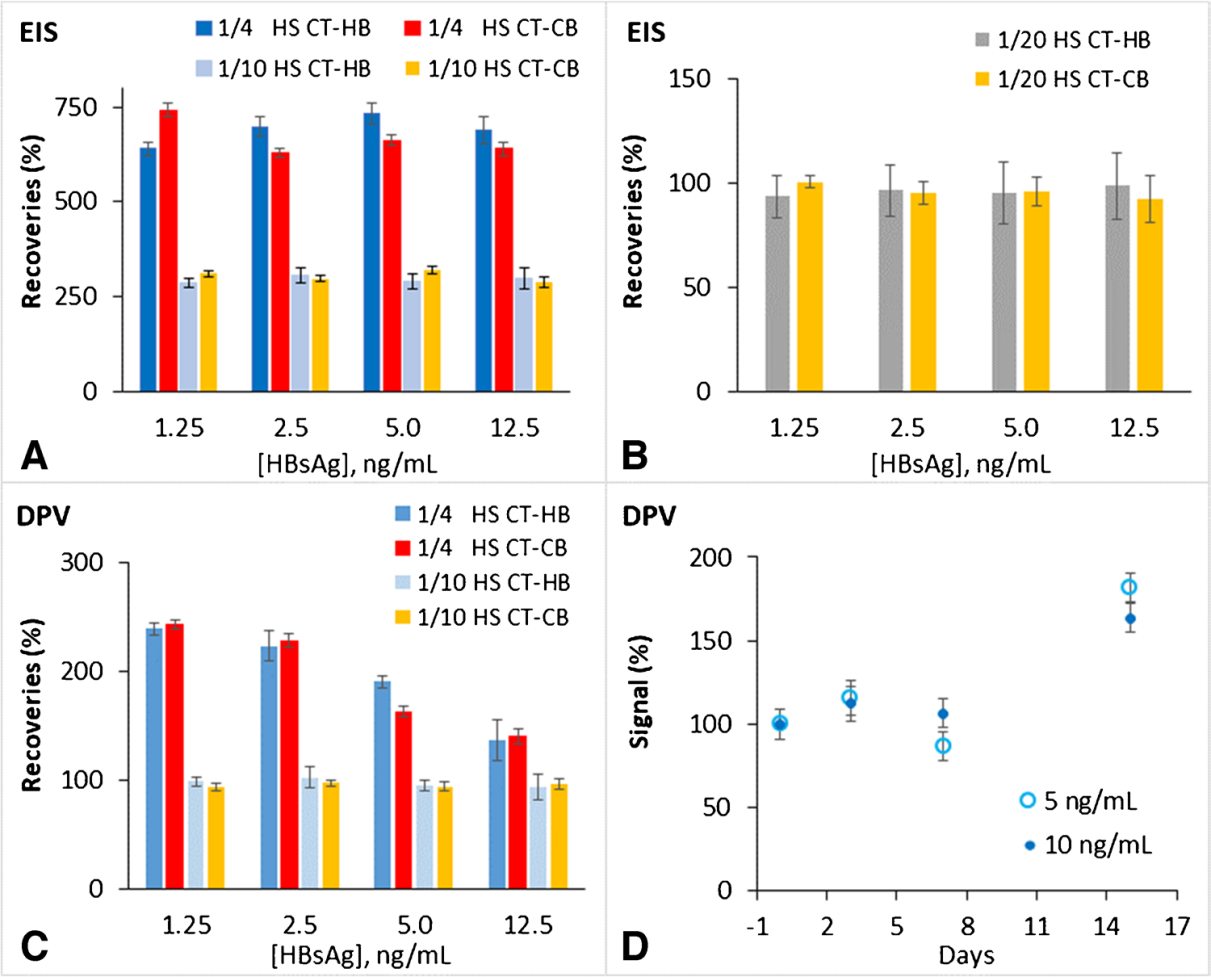
Matrix effect assays in diluted human serum (HS). Percentage of recoveries obtained measuring EIS for CT-HB and CT-CB platforms in 1/4 and 1/10 (**A**) and 1/20 (**B**) human serum/PBS dilutions. **C** Percentage of recoveries obtained measuring DPV for CT-HB and CT-CB platforms in 1/4 and 1/10 human serum/PBS dilutions. **D** Stability assays carried out to the CT-HB biosensor analysing by DPV freshly prepared solutions of 5 and 10 ng/mL HBsAg in 1/10 human serum/PBS dilution

The CT-based biosensors were selected to accomplish the matrix effect assays in diluted human serum because they exhibited better performance than the CS-based counterparts. Initially, 1/4 dilutions were tested to evaluate the recovery percentages. Following, more diluted samples (1/10 and 1/20) were also checked (Figure S7). The results in Fig. 5A–C illustrate that the effect of the human serum matrix depends significantly on the measurement technique used. EIS is more susceptible and more affected by the matrix effect, showing an overestimation of the signal of about 600 and 300% for 1/4 and 1/10 diluted human serum, respectively (Fig. 5 A). Only when a 1/20 dilution was applied, recoveries of around 100% were achieved by EIS for the analysed concentrations (Fig. 5B). However, the same biosensors analysed by DPV were less affected by the serum matrix and exhibited better percentages of recovery of 200 and 100% for 1/4 and 1/10 diluted human serum (Fig. 5C). This difference in the sensor response to the matrix effect is attributed to the fact that EIS is more sensitive to changes occurring on the electrode surface and, hence, the presence of molecules from the serum might affect more the measurements.

The stability and sensing capability of the CT-HB biosensor was assessed after 0, 3, 7 and 15 days of its preparation (Fig. 5D). As observed, after the 3rd and 7th day, the biosensor conserved about 100% of its initial sensing response, indicating that it can be used after 7 days of its preparation to detect the target biomarker in 1/10 diluted human serum. This signal response preservation of the prepared CT-HB biosensor, in addition to demonstrate its potential utility for real sample analysis, highlights the stability of hydrogen bonding for Ab immobilization, as well as, the reliability of this methodology, as an economic and fast alternative for gold surface biomodification.

## Conclusions

Four immunosensors were developed to detect HBsAg in totally label-free assays without adding electron transfer mediators at the electrode surface, using CT or CS as gold surface linkers, and hydrogen bonding or covalent bond for Ab anchoring. The electrical response was evaluated both by DPV and EIS.

The new methodology of combination of HB for Ab immobilization and DPV for electrochemical measurements produced biosensors with improved analytical results, and a simplified strategy for analyte detection, compared to the traditional approach of Ab anchoring by covalent bond and EIS technique:

- This novel approach exhibits larger linear analytical range, better repeatability, less serum matrix effect and similar LoD and LoQ than the Ab immobilization by covalent bond and EIS technique. Moreover, it preserves about of 100% of its biosensing capability after 7 days of the biosensor fabrication.
- The HB Ab immobilisation represents an appealing alternative route to the conventional Ab covalent bond immobilisation. It is a more economic, simpler and faster option than covalent bond for antibodies immobilization, eliminating the reagents used for producing the covalent bonds and reducing steps, time of analysis and costs in general.
- DPV compared to EIS reduces the measurement times and facilitates the data processing.
- Remarkably, an HBsAg label-free biosensor for HBV detection with excellent performance has been demonstrated following the here-in-reported methodology.

HBsAg has been selected as a model protein because of the clinical relevance of this analyte for the world health. However, the reported methodology is general and versatile, which makes it highly promising for further successful implementation in others’ label-free electrochemical assays suitable for POCD biosensors.

### Associate content

Supporting information file is provided with the following information: CV profiles of the optimized gold cleaning procedure; height profiles extracted from AFM topological images; CV, DPV and EIS during each stage of the biofunctionalization process for CS-based biosensors; XPS at each stage of the biomodification process for the CS-CB biosensor; table with the summary of the BE, areas and FWHM in the S 2p, C 1 s and N 1 s regions; EIS and DPV profiles of the CT-CB biosensor over the Ag concentrations tested, table with the Randles circuit parameters during the analytical detection with CT-HB biosensor, and summary tables with the analytical parameters of all electrochemical biosensors developed and others already reported methods for HBsAg detection.

## Supplementary information

Below is the link to the electronic supplementary material.

Supplementary file1 (PDF 1806 KB)

## Data Availability

Data available upon request to the authors.
