# Supplementary material for: Bioreceptors’ immobilization by hydrogen bonding interactions and differential pulse voltammetry for completely label-free electrochemical biosensors
Source: Mikrochim Acta. 2024 Oct 14;191(11):669. doi: 10.1007/s00604-024-06738-x (PMC11473665; doi:10.1007/s00604-024-06738-x)
Supplement: Supplementary file 1 — Supplementary file1 (PDF 1806 KB) [file 604_2024_6738_MOESM1_ESM.pdf]

SUPPORTING INFORMATION

Bioreceptors' immobilization by hydrogen bonding interactions and differential pulse voltammetry for completely label-free electrochemical biosensors

Adaris López_Marzo[a] and Marta Mas-Torrent [a,b*]

[a]Institut de Ciencia de Materials de Barcelona (ICMAB-CSIC), Campus de la UAB, 08193 Bellaterra, Spain; [b] Networking Research Center on Bioengineering Biomaterials and Nanomedicine (CIBER-BBN), 08193 Bellaterra, Spain. *Corresponding author: mmas@icmab.es

A clean gold surface presents a typical CV in sulphuric acid with an intense and characteristic reduction peak at about 0.9 V and three oxidation peaks between 1-1.3 volts that emerge perpendicular to the X axis [1–4]. This characteristic CV for a clean gold surface was achieved for the cleaning method described in section 2.3 of material and methods. This methodology displayed a good repeatability for different gold electrodes and days (Figure S1).

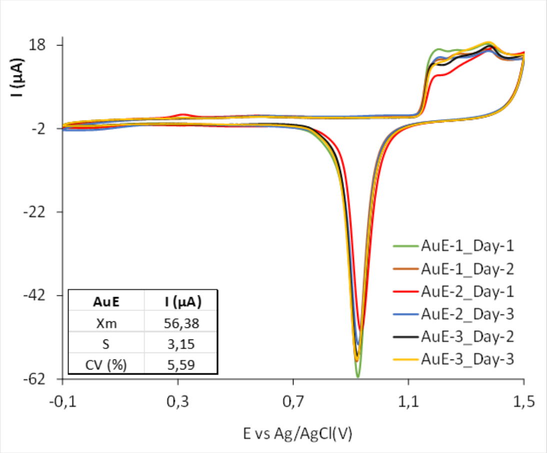

Fig. S1. CVs in H_2SO_4 0.5 M of different gold electrodes (AuE-1, AuE-2, and AuE-3) after the application of optimized cleaning procedure during different days (Day-1, Day-2, and Day-3). In the inset, the evaluation of the repeatability of the optimized cleaning procedure through the coefficients of variation obtained by CV (height of gold reduction peak) using different AuEs over several days.

The real area (electrochemical surface area) of the gold electrodes treated by the optimized procedure was of 0.141 ± 0.012 cm^2 (n = 6) and the roughness factor of 4.50. Both values, the real area and the roughness factor, agree with others reported previously, and were calculated as follow [2, 5]:

$$Q = \frac{A_{Au\ Red\ peak}}{v}$$

$$A_{real} = \frac{Q}{390}$$

$$R_f = \frac{A_{real}}{A_{geom}}$$

Q: charge of the gold reduction peak (μC)

$A_{Au\ red\ peak}$: area of the Au reduction peak (μA)

v : scan rate (0.1 V/s)

A_{real}: area real

390: is a theoretical value for polycrystalline gold electrode (μC/cm^2)

R_f: roughness factor

A_{geom}: area geometrical

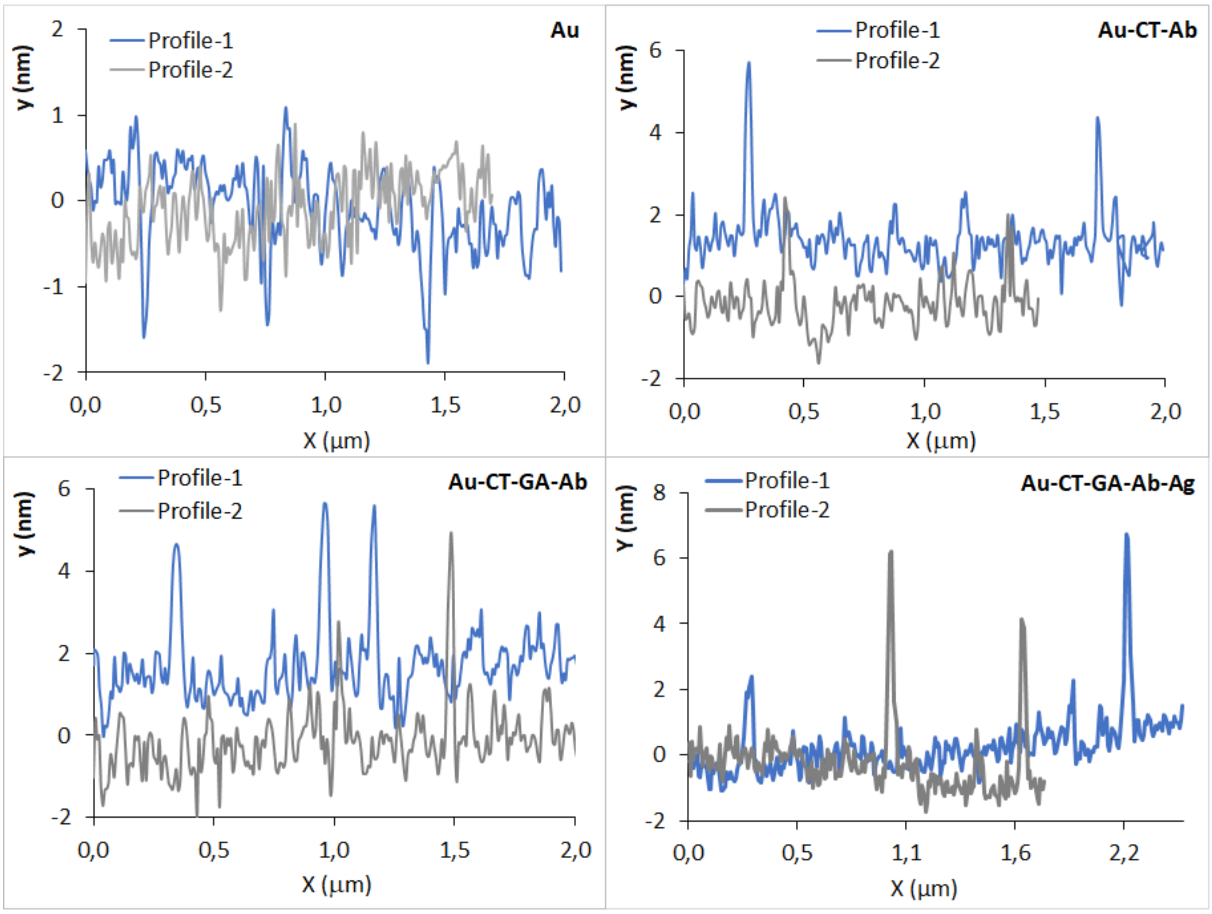

Fig. S2. Profiles of heights obtained from AFM topological images for Au, Au-CT-Ab, Au-CT-GA-Ab and Au-CT-GA-Ab-Ag surfaces at the imaginary lines labelled in Fig. 2.

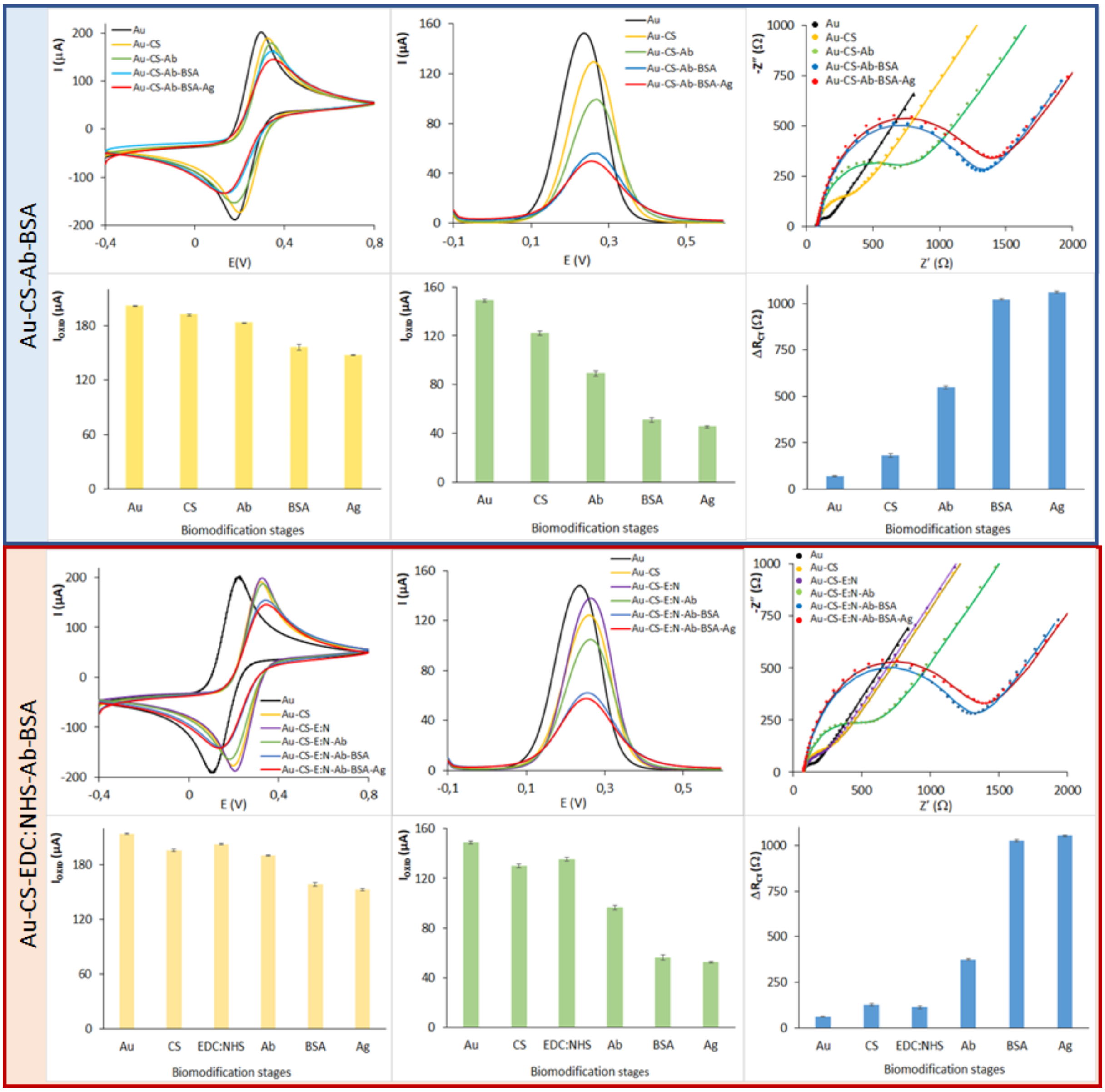

Fig. S3. Electrochemical characterization by CV, DPV and EIS of each stage of the biofunctionalization process for the biosensors using cysteine (CS) as molecular spacer. The CV, DPV and EIS curves are plotted together with their most relevant electrochemical parameters (in the graphs' legend E:N means EDC:NHS). The antigen concentration used was 1.25 ng/mL.

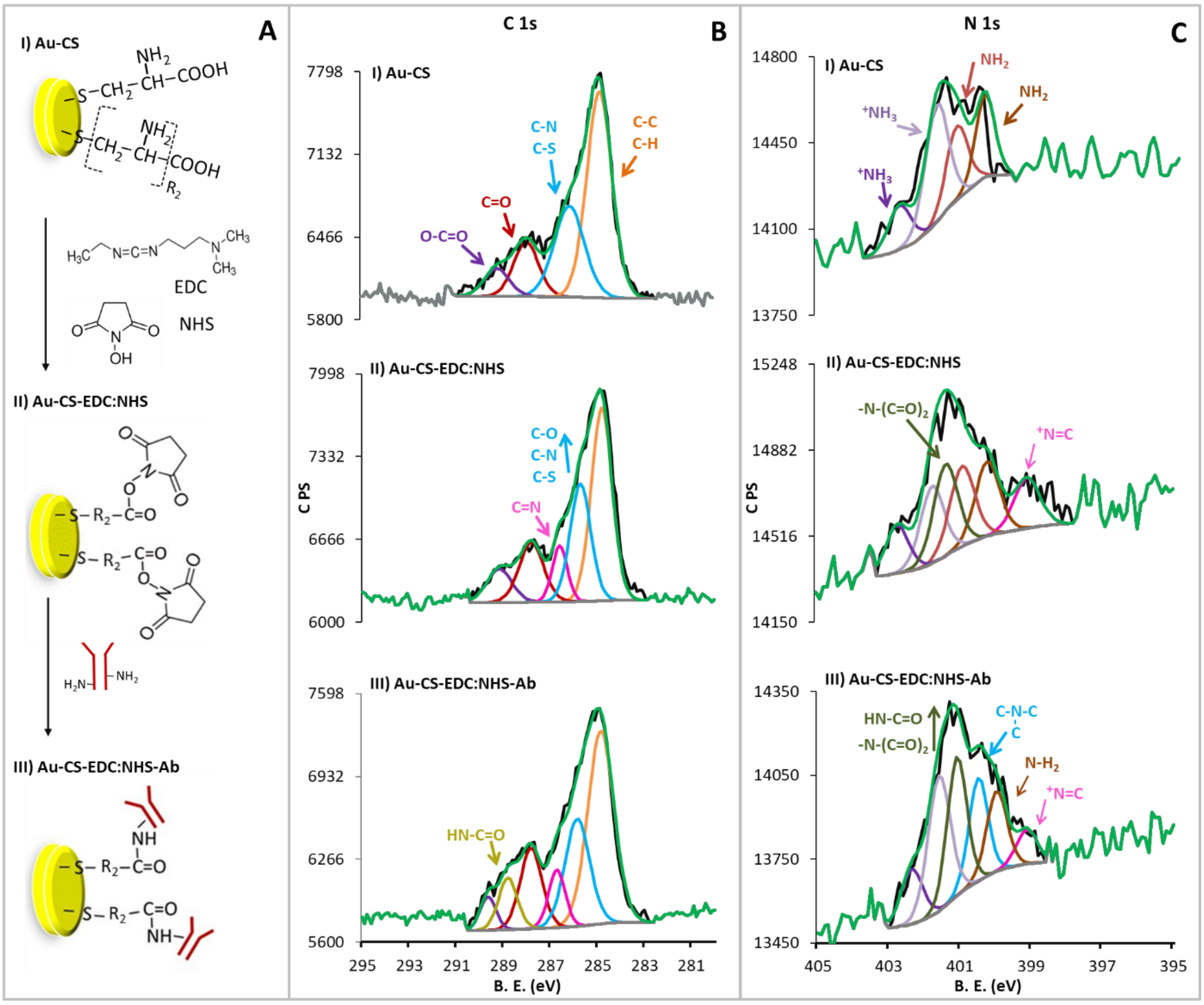

Fig. S4. A) Chemical structure of the Au surface at each stage of the bio-modification process for the Au-CS-EDC: NHS-Ab system and its high-resolution XPS spectra of the C 1s (B) and N 1s (C) core levels.

The XPS study of the bio-modification of the Au-CS-based substrates is illustrated in **Fig. S4**. The main reactions and chemical structure of the compounds at each stage of the bio-functionalization are exhibited in **Fig. S4 A**. In the C 1s region of the Au-CS, four peaks can be identified at 284.8, 286.1, 288 and 289.2 eV which are associated to C-C/C-H, C-N/C-S, C=O and HO-C=O groups, respectively (**Fig. S4 B, I**). After the activation of the COOH groups of the Au-CS with EDC: NHS, the formation of the succinimide takes place and, accordingly, a new peak at 286.6 eV appears related to C=N bonds of EDC residues or resonant structures of succinimide (**Fig. S4 B, II**). The Ab immobilization produced a significant boost in the carbon region at high binding energies, which is associated with an increase in the amount of carbon atoms from the carboxylic and amide groups of the Ab structure (**Fig. S4 B, III**). In the N 1s core level of the Au-CS, similarly to Au-CT surface, the observed region can be deconvoluted into four peaks corresponding to NH_2 and $^{+}NH_3$ with different surroundings (**Fig. S4 C I**). After the reaction with EDC: NHS, the N 1s area is increased because of new contributions of the -N-(C=O)$_2$

succinimide group at 401.4 eV and the –C=N at 399.1 eV coming from EDC residues (**Fig. S4 C, II**). After the Ab anchoring, a notable increase in the peak intensities at high energies of the N 1s region is displayed. As indicated by the augmentation of the signal at 401.6 eV, as well as, by the raising of a new component at 400.5 eV, due to the numerous amides groups and tertiary amines forming the amino acids of the Ab (**Fig. S4 C, III**).

Table S1. Summary of the peaks' assignations and their binding energies (B. E. in eV), areas (in %) and full width at half maximum (FWHM, in eV) in the S 2p core level of the HR-XPS for Au-CT and Au-CS substrates [2, 6, 7].

**S 2p**	**Au-CT**						**Au-CS**					
	2p 3/2			2p 1/2			2p 3/2			2p 1/2		
	B. E.	Area	FWHM	B. E.	Area	FWHM	B. E.	Area	FWHM	B. E.	Area	FWHM
S-Au	161.29	11.81	0.64	162.29	12.88	0.66	161.15	16.73	0.70	162.29	10.97	0.62
S-Au	162.05	31.21	0.67	162.90	16.65	0.67	161.98	29.69	0.67	162.68	16.04	0.67
S-H	163.42	16.89	0.66	164.14	10.31	0.63	163.35	17.61	0.66	164.10	8.97	0.61
S-Au / S-H	2.7 (72.8 %)						2.8 (73.4 %)					

Table S2. Summary of the bond assignation and their binding energies (in eV) in the C 1s core level of the HR-XPS for Au-CT and Au-CS based substrates [8–12].

**C 1s**					
C-H; C-C	-C-N; -C-S -C-OH	C=N	C=O	N-C=O	O-C=O
285.0 ± 0.2	285.9 ± 0.2	286.8 ± 0.2	287.9 ± 0.1	288.7 ± 0.1	289.4 ± 0.2

Table S3. Summary of the peaks' assignations and their binding energies (B. E. in eV), areas (in %) and full width at half maximum (FWHM, in eV) in the N 1s core level of the HR-XPS for Au-CT and Au-CS based substrates. (*) means groups with different surroundings [8–11, 13, 14].

N 1s		N=C	$^{+}$N=C	C-NH_2	*C-NH_2	-N-(C=O)$_2$	C-$^{+}NH_3$	*C-$^{+}NH_3$
**Au-CT**	B. E.	-	-	399.22	400.21	-	401.20	402.25
	Area	-	-	42.69	23.49	-	20.37	13.45
	FWHM	-	-	1.20	1.20	-	1.10	1.10
**Au-CT-GA**	B. E.	397.90	398.60	399.39	400.20	-	401.22	402.25
	Area	6.53	21.38	23.09	13.51	-	12.65	22.85
	FWHM	0.80	0.85	0.90	0.85	-	0.90	0.92
**Au-CS**	B. E.	-	-	400.34	401.15	-	401.67	402.70
	Area	-	-	23.13	23.05	-	39.17	14.65
	FWHM	-	-	0.63	0.70	-	0.75	0.75
**Au-CS-EDC:NHS**	B. E.	-	399.09	400.20	400.90	401.40	401.74	402.73
	Area	-	12.73	17.70	19.28	21.62	17.77	10.90
	FWHM	-	0.85	0.80	0.81	0.79	0.78	0.76

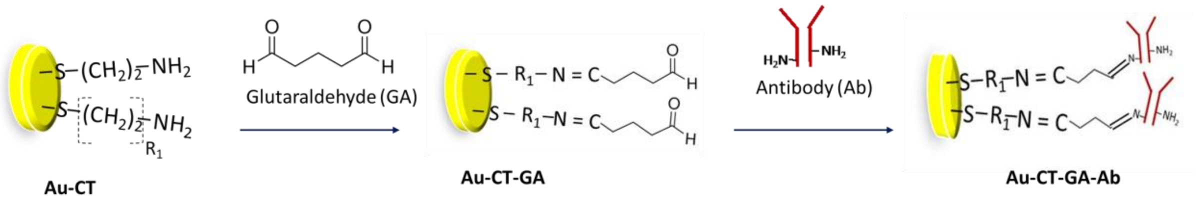

Fig. S5. Chemical structure of the SAM on Au surface at each stage of the biomodification process for the Au-CT-GA-Ab system.

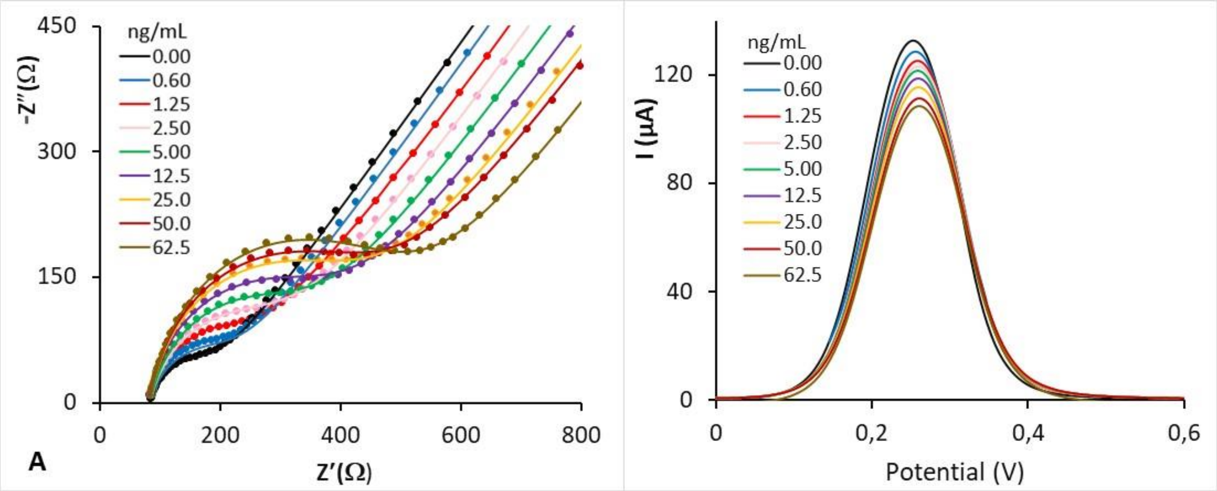

Fig. S6. Analysis of standard solutions of increasing HBsAg concentrations using the CT-CB biosensor by EIS (A) and DPV (B) techniques.

Table S4. Parameters of the Randles circuit calculated during the HBsAg detection using the CT-HB biosensor. The Rs is the solution resistance, R_{CT} is the charge transfer resistance, Qdl the constant-phase-element, W the Warburg impedance, and χ^2 the convergence factor.

Ag (ng/mL)	Rs (Ω)	R_{CT} (Ω)	Q µMho*s$^{0.85}$	W µMho*s$^{1/2}$	χ^2
0.00	81.00	226.80	5.06	208.60	0.0086
0.60	80.81	250.05	4.64	216.20	0.0062
1.25	80.60	297.00	4.26	214.00	0.0077
2.50	80.15	345.03	4.15	211.82	0.0100
5.00	81.22	457.61	3.14	208.85	0.0122
12.5	81.44	521.29	2.99	202.31	0.0190
25.0	81.71	639.63	2.53	224.02	0.0330
50.0	82.28	739.85	2.42	226.13	0.0250
62.5	82.36	838.35	2.32	206.73	0.0182

Table S5. Analytical parameters of the four-immunosensors studied by EIS and DPV techniques. In all the biosensor platforms the concentration range studied was 0 - 62.5 ng/mL. The slope of the calibration curve is the sensitivity, S, expressed in Ω·mL/ng or μA·mL/ng for EIS or DPV, respectively. The molecular weight of the Recombinant Hepatitis B Virus Hepatitis B Surface Antigen is 24 kDa.

Biosensors	Technique	Linear Range (ng/mL)	R^2	S	LoQ (ng/mL)	LoQ (pM)	LoD (ng/mL)	LoD (pM)
CT-HB	EIS	0 – 5.0	0.992	41.67	0.43	17.87	0.13	5.42
	DPV	0 – 12.5	0.989	4.14	0.46	19.25	0.14	5.83
CT-CB	EIS	0 – 5.0	0.986	24.72	0.79	33.00	0.24	10.00
	DPV	0 – 5.0	0.980	1.72	1.06	44.00	0.32	13.33
CS-HB	EIS	0 – 5.0	0.999	31.68	0.92	38.50	0.28	11.67
	DPV	0 – 12.5	0.997	3.32	0.83	34.38	0.25	10.42
CS-CB	EIS	0 – 5.0	0.999	20.33	1.25	52.25	0.38	15.83
	DPV	0 – 5.0	0.996	1.18	1.55	64.63	0.47	19.58

Table S6. Other electrochemical immunosensors recently reported for the detection of surface antigen of the HBV (HBsAg). For comparison linear range and LoD values have been converted to molar concentration (M) considering 24 kDa the molecular weight of HBsAg [15–20].

References	Technique Assay format	Working electrode biofunctionalization	Biomarker Ag	Analytical Range (M)	LoD (M)	Matrix analyzed
15	Amperometry Sandwich format	GCE/GO@AuNPs-Ab_1/Ag/ Ab_2-Cu_2OMoS_2-Pt	HBsAg	$2.1x10^{-14} – 8.3x10^{-9}$ *	6.2×10^{-15}	human serum
16	Amperometry Sandwich format	GCE/AuNPs/PPyNS/Ab_1/Ag/ Ab_2-RhPtNDs-NH_2-GS	HBsAg	$2.1x10^{-14} – 4.2x10^{-10}$ *	7.0×10^{-15}	human serum
17	DPV Label-with using AgNPs as electroactive signal and CNT as electron mediator	SPCE/CNT-AuNPs/AgNPs/EDC-NHS/Ab/Ag	HBsAg	$4.2x10^{-11} – 1.6x10^{-9}$	3.6×10^{-11}	human serum 100 times diluted
18	EIS Total label-free	SPCE/BSA /EDC-NHS/Ab/Ag	HBsAg	$2.1x10^{-10} – 1.3x10^{-7}$ *	8.7×10^{-11}	-
19	Amperometry Label-free using AuNPs as electron transfer mediator	SPGE/AuNPs/β-CD/Ab/Ag	HBsAg	$4.2x10^{-7} – 8.3x10^{-6}$ *	7.1×10^{-9}	human serum
20	SWV Sandwich format	GCE/Au@Pt/Ab_1/Ag/Ab_2-GS-SnO_2-Au@Pt	HBsAg	$4.2x10^{-13} – 4.2x10^{-9}$ *	2.0×10^{-13}	human blood
This work	DPV Total label-free	AuE/CysT/Ab/Ag	HBsAg	$19.2x10^{-12} – 5.2x10^{-10}$	$5,8 \times 10^{-12}$	human serum

AgNPs: silver nanoparticles, AuE: gold electrodes, AuNPs: gold nanoparticles, β-CB: beta-cyclodextrin,

CHI: chitosan, GCE: glassy carbon electrode, GO: graphene oxide, GS: graphene sheet, MWCNTs: multiwalled carbon nanotubes, PtE: platinum electrodes, rGO: reduced graphene oxide, RhPtNDs: Rh core and Pt shell nanodendrites, SPCE: screen-printed carbon electrodes, SPGE: screen-printed graphene electrodes. * These linear ranges were achieved fitting to log[HBsAg].

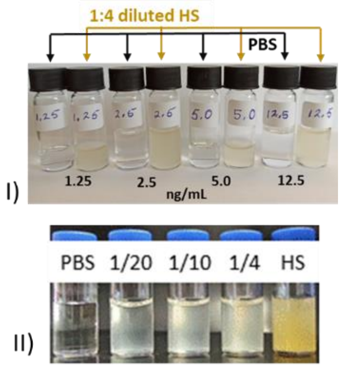

Figure S7. I) Photo of the HBsAg solutions at the 1.25; 2.5; 5,0 and 12.5 ng/mL concentrations in PBS and in 1/4 diluted human serum. II) Photo of PBS and HS at different ratios.

References

1. McCormick W, McDonagh P, Doran J, McCrudden D (2021) Covalent Immobilisation of a Nanoporous Platinum Film onto a Gold Screen-Printed Electrode for Highly Stable and Selective Non-Enzymatic Glucose Sensing. Catalysts 11:1161. https://doi.org/10.3390/catal11101161

2. Makaraviciute A, Xu X, Nyholm L, Zhang Z (2017) Systematic Approach to the Development of Microfabricated Biosensors: Relationship between Gold Surface Pretreatment and Thiolated Molecule Binding. ACS Appl Mater Interfaces 9:26610–26621. https://doi.org/10.1021/acsami.7b08581

3. Jeyabharathi C, Hasse U, Ahrens P, Scholz F (2014) Oxygen electroreduction on polycrystalline gold electrodes and on gold nanoparticle-modified glassy carbon electrodes. J Solid State Electrochem 18:3299–3306. https://doi.org/10.1007/s10008-014-2657-y

4. Ma W, Ying YL, Qin LX, et al (2013) Investigating electron-transfer processes using a biomimetic hybrid bilayer membrane system. Nat Protoc 8:439–450. https://doi.org/10.1038/nprot.2013.007

5. Sukeri A, Saravia LPH, Bertotti M (2015) A facile electrochemical approach to fabricate a nanoporous gold film electrode and its electrocatalytic activity towards dissolved oxygen reduction. Physical Chemistry Chemical Physics 17:28510–28514. https://doi.org/10.1039/C5CP05220C

6. Jürgensen A, Raschke H, Esser N, Hergenröder R (2018) An in situ XPS study of L-cysteine coadsorbed with water on polycrystalline copper and gold. Appl Surf Sci 435:870–879. https://doi.org/10.1016/j.apsusc.2017.11.150

7. Wirde M, Gelius U, Nyholm L (1999) Self-assembled monolayers of cystamine and cysteamine on gold studied by XPS and voltammetry. Langmuir 15:6370–6378. https://doi.org/10.1021/la9903245

8. Šetka M, Calavia R, Vojkůvka L, et al (2019) Raman and XPS studies of ammonia sensitive polypyrrole nanorods and nanoparticles. Sci Rep 9:1–11. https://doi.org/10.1038/s41598-019-44900-1

9. Tong Y, Berdiyorov GR, Sinopoli A, et al (2021) An estimation on the mechanical stabilities of SAMs by low energy Ar + cluster ion collision. Sci Rep 11:12772. https://doi.org/10.1038/s41598-021-92077-3

10. Wang X, Zu X, Wang T, et al (2023) N, S-Doped Carbon Dots Prepared by Peanut Protein Isolates and Cysteamine as Highly Sensitive Fluorescent Sensors for Fe2+, Fe3+ and Lactoferrin. Polymers (Basel) 15:. https://doi.org/10.3390/polym15010216

11. Online VA, Ederer J, Ecorchard P, et al (2017) Determination of amino groups on functionalized graphene oxide for polyurethane nanomaterials: XPS quantitation vs. functional speciation. RSC Adv 7:12464–12473. https://doi.org/10.1039/c6ra28745j

12. Biesinger MC (2022) Accessing the robustness of adventitious carbon for charge referencing (correction) purposes in XPS analysis: Insights from a multi-user facility data review. Appl Surf Sci 597:153681. https://doi.org/10.1016/j.apsusc.2022.153681

13. Min H, Girard-Lauriault PL, Gross T, et al (2012) Ambient-ageing processes in amine self-assembled monolayers on microarray slides as studied by ToF-SIMS with principal component analysis, XPS, and NEXAFS spectroscopy. Anal Bioanal Chem 403:613–623. https://doi.org/10.1007/s00216-012-5862-5

14. Lai LJ, Yang YW, Lin YK, et al (2009) Surface characterization of immunosensor conjugated with gold nanoparticles based on cyclic voltammetry and X-ray photoelectron spectroscopy. Colloids Surf B Biointerfaces 68:130–135. https://doi.org/10.1016/j.colsurfb.2008.09.010

15. Li F, Li Y, Feng J, et al (2018) Facile synthesis of MoS2@Cu2O-Pt nanohybrid as enzyme-mimetic label for the detection of the Hepatitis B surface antigen. Biosens Bioelectron 100:512–518. https://doi.org/10.1016/j.bios.2017.09.048

16. Pei F, Wang P, Ma E, et al (2019) A sandwich-type electrochemical immunosensor based on RhPt NDs/NH2-GS and Au NPs/PPy NS for quantitative detection hepatitis B surface antigen. Bioelectrochemistry 126:92–98. https://doi.org/10.1016/j.bioelechem.2018.11.008

17. Upan J, Banet P, Aubert P-H, et al (2020) Sequential injection-differential pulse voltammetric immunosensor for hepatitis B surface antigen using the modified screen-printed carbon electrode. Electrochim Acta 349:136335. https://doi.org/10.1016/j.electacta.2020.136335

18. Akkapinyo C, Khownarumit P, Waraho-Zhmayev D, Poo-Arporn RP (2020) Development of a multiplex immunochromatographic strip test and ultrasensitive electrochemical immunosensor for hepatitis B virus screening. Anal Chim Acta 1095:162–171. https://doi.org/10.1016/j.aca.2019.10.016

19. Teengam P, Siangproh W, Tontisirin S, et al (2021) NFC-enabling smartphone-based portable amperometric immunosensor for hepatitis B virus detection. Sens Actuators B Chem 326:128825. https://doi.org/10.1016/j.snb.2020.128825

20. Jiang L, Li Y, Xu Z, et al (2021) Simultaneous electrochemical determination of two hepatitis B antigens using graphene-SnO_2 hybridized with sea urchin–like bimetallic nanoparticles. Microchimica Acta 188:109. https://doi.org/10.1007/s00604-021-04763-8
